# Molecular Evidence of Polyandry in the Citrus Mealybug, *Planococcus citri* (Hemiptera: Pseudococcidae)

**DOI:** 10.1371/journal.pone.0068241

**Published:** 2013-07-03

**Authors:** Sofia G. Seabra, Patricia G. Brás, Vera Zina, Elsa Borges da Silva, Maria Teresa Rebelo, Elisabete Figueiredo, Zvi Mendel, Octávio S. Paulo, José Carlos Franco

**Affiliations:** 1 Computational Biology and Population Genomics Group, Centro de Biologia Ambiental, Departamento de Biologia Animal, Faculdade de Ciências da Universidade de Lisboa, Lisboa, Portugal; 2 Centro de Estudos Florestais, Instituto Superior de Agronomia, Universidade Técnica de Lisboa, Lisboa, Portugal; 3 Centro de Estudos do Ambiente e do Mar, Departamento de Biologia Animal, Faculdade de Ciências da Universidade de Lisboa, Lisboa, Portugal; 4 Centro de Engenharia dos Biossistemas, Instituto Superior de Agronomia, Universidade Técnica de Lisboa, Lisboa, Portugal; 5 Department of Entomology, Agricultural Research Organization, the Volcani Center, Bet Dagan, Israel; University of Sussex, United Kingdom

## Abstract

The occurrence of polyandry in *Planococcus* *citri* , presumed by earlier observations of mating behavior, was confirmed using microsatellite genotyping of pools of over 400 eggs resulting from controlled crosses of one female with two males. The genetic contribution of both mated males was confirmed in 13 out of 43 crosses. In three crosses it was possible to determine that only the first male fertilized the eggs, which may be due to sperm competition or unviable sperm supply. The microsatellite analysis also allowed the confirmation of aspects of the chromosomal inheritance detected previously in cytogenetic studies in *Planococcus* *citri* , namely that only one of the alleles is transmitted by the male, indicating that the males are functionally haploid, supporting the observation of Paternal Genome Elimination (PGE) in these insects.

## Introduction

Polyandry is a widespread phenomenon in insects and may have significant fitness consequences for both sexes [1]. The act of mating can carry considerable costs to females including time and energy costs, as well as increased exposure to disease or predation, but these costs may be overcome by both direct and indirect benefits [1–3]. Direct benefits of polyandry include replenishment of depleted or unviable sperm supplies, the transfer of nuptial gifts and nutrients, access to resources, and protection from male harassment, whereas indirect genetic benefits are related to increased viability and/or reproductive success of the progeny [2] (and references therein). Polyandry also affects fitness of males since the sperm of different males will have to compete for successful fertilizations [4].

Polyandry might have evolved as a result of selection against selfish genetic elements [5–7]. On the other hand, speciation is apparently influenced by polyandry. It was shown that polyandrous insect lineages have a much higher speciation rate in comparison with related monandrous lineages, as a result of postmating sexual conflict, as well as postmating sexual selection [8].

Mealybugs (Hemiptera, Pseudococcidae) constitute the second largest family of scale insects (Coccoidea) and include many economic important pest species of different crops worldwide [9,10]. The males of mealybugs are polygynic, being capable of fertilizing multiple females during their short lifespan ( [10,11]; Silva et al., unpublished data). Almost no information is available on the literature on the mating system of mealybug females, although they are presumed to be monandrous. However, recent behavioral experiments showed that virgin mealybug females of *Pseudococcus* *longispinus* (Targioni-Tozzetti), *Pseudococcus* *viburni* (Signoret), *Pseudococcus* *maritimus* (Ehrhorn), *Planococcus* *ficus* (Signoret) [11,12], *Pseudococcus* *calceolariae* (Maskell), and *Planococcus* *citri* (Risso) ( [13]; Silva et al., unpublished data) remain receptive several days after the first mating and eventually can mate multiple times with the same or different males, suggesting the existence of polyandry in these insects. Nevertheless, the involvement of more than one male in the fertilization of the eggs oviposited by multiply-mated mealybug females was not demonstrated in those studies. In fact, in many insect species, the sperm from the most recent male to copulate is more likely to fertilize the female’s eggs [14,15]. Here, we present for the first time genetic evidence for the occurrence of polyandry in mealybugs, using as a model the citrus mealybug, *Planococcus* *citri* , one of the most economically important mealybug species worldwide. It is a pest mainly of subtropical fruit crops in Mediterranean regions and of ornamental plants in interior landscapes [10]. Besides the theoretical interest, investigating the hypothesis of polyandry in mealybugs has also practical implications in pest management. When designing control programmes of insect pests it is crucial to know if females mate with a single male or with multiple males [14,16].

The analysis of parentage in mealybugs constitutes a very particular situation due to the patterns of chromosome inheritance and sex determination system of these insects. In Lecanoid Paternal Genome Elimination (PGE), the genetic system of mealybugs, the sex determination mechanism involves the inactivation by heterochromatization of one haploid set of chromosomes in males, whereas in females both sets are active [17]. The inactivated set of chromosomes in males is of paternal origin [18]. It is eliminated during spermatogenesis, and therefore only the maternally-derived euchromatic chromosomes are transmitted by males to the progeny [17,19]. Mealybug males are thus functionally haploid, making this system in evolutionary terms analogous to haplodiploidy and to sex-linked loci in diploid animals [19,20]. However, the mechanism of sex determination in PGE system is still unknown. Mealybug sex may be determined either by facultative imprinting or by proteins of maternal origin which are added to the eggs (see 21 for a review).

Although detailed cytogenetic studies have been done in mealybugs and have revealed the patterns of inheritance described above, almost no genetic evidence is available [22]. The analysis of highly polymorphic genetic markers, such as microsatellites [23–25] can be used for that purpose. We explored this approach, based on the microsatellites recently developed for *P* *. citri* [26], aiming to test the hypothesis of polyandry in mealybugs, suggested in recent behavioral studies, by providing genetic evidence of multiple paternal contribution to the progeny of multiply-mated females. Genetic evidence that mealybug males only contribute half of their genetic material to the progeny, since their haploid set of chromosomes of paternal origin is expected to be eliminated during spermatogenesis, is also presented. In *P* *. citri* , the progeny of each cross consists of a high number of embryos of both sexes (average of 467 eggs oviposited per female at 25^°^C, 70% RH and photoperiod of 12L:12D, when feeding on sprouted potatoes [27]). As genotyping every single individual from the progeny of several crosses is costly and time consuming, we used entire pools of eggs, which turned out to be an efficient approach in the detection of polyandry in this species.

## Materials and Methods

### Origin and rearing of mealybugs

The mealybugs used in the bioassays were collected from sweet orange (*Citrus sinensis* (L.)) trees, and reared in climatic chambers (24±0.5 °C, 60% RH, in total darkness) inside plastic containers with sprouted potatoes (*Solanum tuberosum* L.). In order to maximize the allele diversity between parents, different geographical origins were used: Agualva, Camarate, Mafra, Silves and Tavira, from Portugal, and Xirumi-Serravalle, from Italy. Field collected individuals were periodically added to the laboratory colonies, in order to refresh the rearing.

### Ethics Statement

The field sampling was carried out on private lands with owners’ permissions. The studied species, *Planococcus* *citri* , is considered a common insect pest of citrus and other crops in the Mediterranean area and is not an endangered or protected species.

### Mealybug crosses

The mating bioassays were set up in Petri dish arenas under laboratory conditions. Virgin males and females were obtained by isolating, in plastic containers, prepupae and third instar nymphs, respectively, as described in [13]. Each female was exposed to a sexually mature male, i.e., at least 29 hours old [28]. After copulation, the male was withdrawn, and a second male was introduced and kept in the arena until a second mating had occurred. Mealybug mating was confirmed by direct observation under magnification (x 30~50; Nikon SMZ-2B, Japan), with the help of a mirror installed beneath the Petri dish. After double mating, females were kept in Petri dishes with a food supply, for ovipositing. Laid eggs were daily collected and preserved in ethanol till the end of the oviposition period. For each cross, the two males, corresponding female and egg mass were kept separately in ethanol inside labeled Eppendorf tubes, for later DNA extraction and analysis.

In total, 43 crosses were carried out for the analysis (Table 1). The mealybug specimens of each cross were genotyped for six microsatellites developed earlier for *P* *. citri* [26].

**Table 1:** Number of alleles and observed and expected heterozygosities (H_o_ and H_e_, respectively) for each microsatellite locus in the 139 parents (females and males) of *Planococcus* *citri* used in the mating experiments.

**Locus**	**Number of alleles**	**H_o_**	**H_e_**
Pci-6	4	0.352	0.540
Pci-7	2	0.420	0.398
Pci-14	3	0.302	0.321
Pci-16	5	0.512	0.698
Pci-17	3	0.089	0.366
Pci-20	2	0.112	0.281

### DNA extraction and microsatellite genotyping

DNA extraction was done with E.Z.N.A. Tissue DNA Isolation kit (Omega) following the manufacturer’s protocol. Amplification of microsatellite loci was performed as detailed in [26] using the M13-tailed primer protocol for fluorescence labelling of PCR fragments [29]. Microsatellites were genotyped in an ABI PRISM 310 Genetic Analyser (Applied Biosystems) with GeneScan Rox Size Standard (Applied Biosystems) as internal size standard. Microsatellite loci were scored using GeneMapper v4 (Applied Biosystems). Numbers of alleles, and expected and observed heterozygosities in the parents were obtained with GENETIX v 4.05.2 [30]. Statistical tests for the comparisons of polymorphism and heterozygosity were done using R version 2.15.0 [31]. Deviations from Hardy-Weinberg equilibrium were tested using GENEPOP 4.1.2 [32]. The application of the Hardy-Weinberg principles to the case of haplodiploid systems indicates that the allele frequencies in males and females will oscillate around the mean frequency and will converge to that value after a few generations of random mating [20], in contrast to what happens in a diploid system in which an equilibrium is reached after only one generation of random mating and in which no differences in allele frequencies between sexes are expected. Different allelic frequencies in the two sexes in the haplodiploid system leads to an excess of heterozygotes compared to Hardy-Weinberg proportions [20]. PGE, the genetic system of mealybugs differs from arrhenotokous haplodiploidy, in which the males develop from unfertilized eggs and have only a haploid maternal genome (e.g., Thysanoptera, Hymenoptera, Hemiptera, and Coleoptera). Mealybug males develop from diploid zygotes and are functionally haploid in the sense that only the maternal genome is transmitted to the progeny [19]. Therefore, we expect to detect the diploid genotypes of microsatellite loci in *P* *. citri* males, and differently from the haplodiploid case, the allele frequencies of males and females at each generation are not expected to be different, assuming random mating, random gamete fusion and random sex determination.

**Figure 1:**
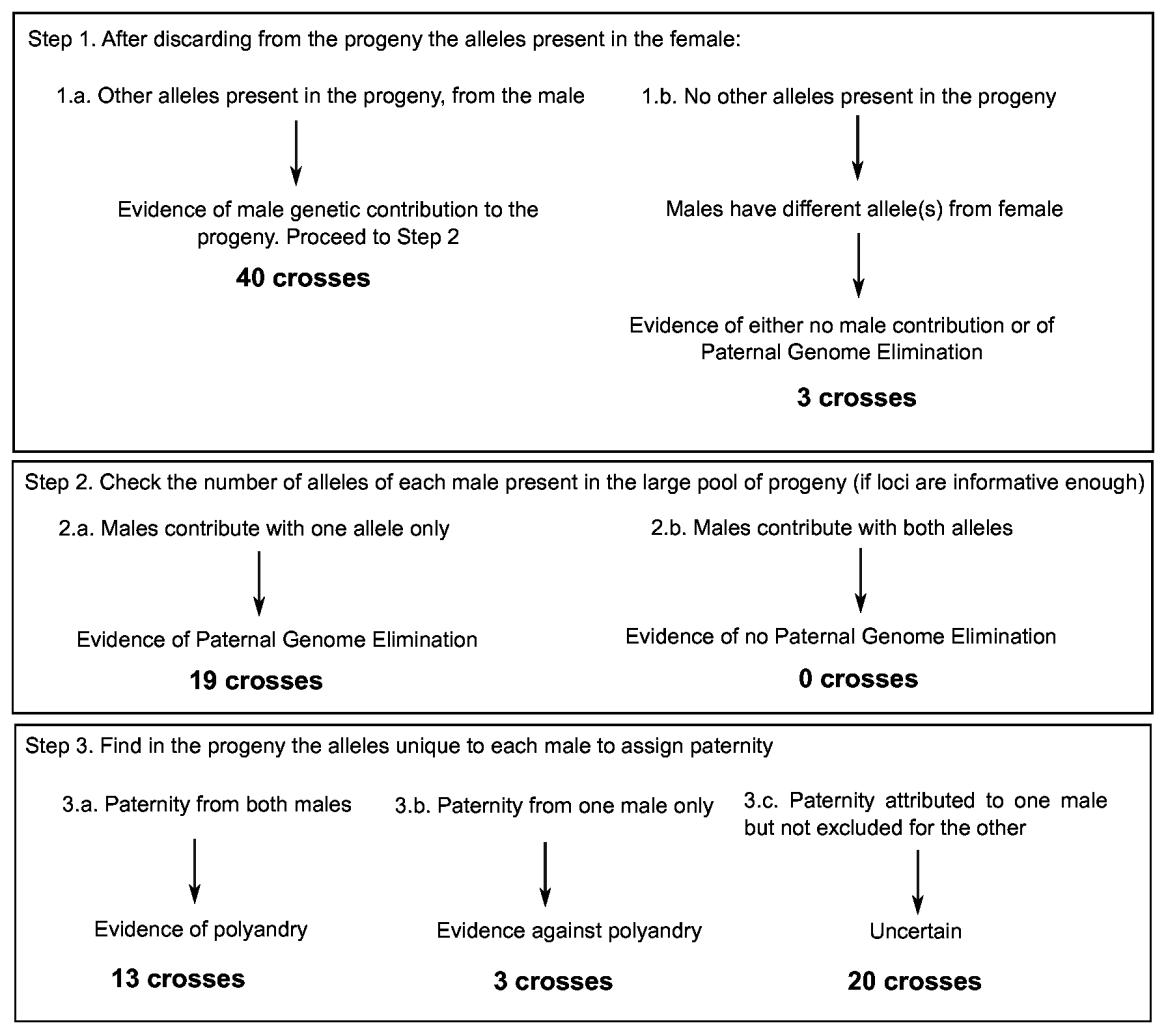
Flow chart of the analysis of polyandry in *Planococcus* *citri* controlled crosses.

Most approaches available for parentage analysis are based on the assumption of mendelian segregation of the alleles and in the estimation of allele frequencies of the population under study [33,34]. In our system and methodological approach, we should consider that a) a pool of eggs is used instead of single-individual egg genotypes, b) the male is expected to transmit through his sperm only the maternal half of the genome, due to paternal genome elimination in spermatogenesis, and c) parents from different source populations are selected. The flow-chart designed for the analysis is shown in Figure 1. Since in this experimental scenario the female and males of each cross are known, the assignment of parentage is easier than in natural conditions where usually the male parent and sometimes also the female parent is unknown, and where parentage is assigned as a likelihood or posterior probability value [34].

## Results

The number of alleles at each locus ranged from 2 to 5, and the observed and the expected heterozygosity, calculated only from the parents, ranged from 0.089 to 0.512 and from 0.281 to 0.698, respectively (Table 1). F_IS_ were generally high (> 0.2) and significant (<0.0001) for all loci except for Pci-7 and Pci-14, for which F_IS_ were near zero and non-significant. These deviations in Hardy-Weinberg equilibrium indicate a significant deficit of heterozygotes which are likely caused by substructure due to the different geographic origins of the parents (Wahlund effect).

The loci with higher numbers of alleles (Pci-6 and Pci-16) were the ones that allowed single-locus detection of polyandry. The other loci generally allowed attribution of paternity to one father but were not informative concerning the other one (Table 2).

**Table 2:** Alleles of the crosses that allowed the detection of polyandry in *Planococcus* *citri* in each cross.

**Cross**	**Locus**	**Female alleles**	**First male alleles**	**Second male alleles**	**Progeny**
*Detection of polyandry*					
Cross 9	Pci-16	206	206/**215**	206/**218**	206/**215**/**218**
Cross 65	Pci-16	212/215	**206**/215	**218**	**206**/212/215/**218**
Cross 55	Pci-16	215/218	**212**	**206**/215	**206**/**212**/215/218
Cross 79	Pci-16	218	**212**	**206**	**206**/**212**/218
Cross 86	Pci-16	215/218	**212**	**206**	**206**/**212**/215/218
Cross 93	Pci-6	159	144/**171**	159/**177**	159/**171**/**177**
	Pci-16	215/218	**212**/215	**206**	**206**/**212**/215/218
Cross 96	Pci-16	206	**212**	206/**215**	206/**212**/**215**
Cross 101	Pci-6	159	144/**171**	159	159/**171**
	Pci-16	206	**212**/215	206/215	206/**212**/215
	Pci-17	220	211/**217**	220	**217**/220
	Pci-20	253	253	**256**	253/**256**
Cross 102	Pci-6	159	**171**	159/**177**	159/**171**/**177**
	Pci-17	220	**217**	220	**217**/220
	Pci-20	253	253	**256**	253/**256**
Cross 108	Pci-6	159	**171**	159	159/**171**
	Pci-17	220	**211**	220	**211**/220
	Pci-20	253	253	253/**256**	253/**256**
*Detection of polyandry assuming Paternal Genome Elimination*					
Cross 66	Pci-6	171	159/**177**	144/**159**	**159**/171/**177**
Cross 68	Pci-16	212	206/**215**	**206**	**206**/212/**215**
Cross 109	Pci-6	171	**159**	159/**177**	**159**/171/**177**

Alleles of the males detected in the progeny are in bold.

The number of eggs in each batch laid by one female is very high and we assume that every possible combination of alleles from the parents will appear in the pool of eggs. The analysis of parentage of each cross was done by looking at the alleles of every locus following the scheme on Figure 1. After discarding from the progeny the alleles observed in the female, in 40 crosses out of the 43 crosses analysed, other alleles were detected in the eggs (the same observed in one or two of the male parents), indicating male genetic contribution to the progeny. In the remaining three crosses, one of the males had a different allele from the female, at least at one locus, which was not detected in the progeny, evidencing either no male contribution or PGE. In the 19 crosses where males were heterozygous at one or more loci, and contributed to the offspring, no male was detected transmitting both alleles, which is an indication of PGE.

From the studied 43 mealybug crosses, 13 showed evidence of polyandry, through the presence of alleles unique to each male in the same or in different loci. In three of the cases, evidence of polyandry is based on the assumption of PGE; that is, only one allele could come from each male (Table 2).

In 20 other crosses we could attribute the paternity to the first male and could not exclude it for the second male. The reverse situation was not found in any of the crosses. We examined whether this could be due to higher polymorphism and heterozygosity in the first males than in the second males, by chance in the choice of male order during the setup of the crosses. Indeed the average number of alleles and the average observed and expected heterozygosities were higher in the first males than in the second ones (Table 3), although the differences were not significant for the number of alleles (Wilcoxon signed-rank test, p=0.1736) or the observed heterozygosity (Wilcoxon signed-rank test, p=0.1563) and were significant for the expected heterozygosity (Wilcoxon signed-rank test, p=0.0312). The slight difference may be explained by the different origins of the populations chosen to provide first and second males. The microsatellite variability found in the parents from different populations revealed higher polymorphism and/or heterozygosity in some of the populations, namely Agualva and Xirumi-Serravalle in comparison with Mafra and Silves (Table 3), although Wilcoxon signed-rank tests were not significant in any paired comparisons. The values from Camarate and Tavira are not comparable since the sample sizes are very small. First males were mainly from Agualva and Xirumi-Serravalle and second males were from Agualva, Mafra and Silves.

**Table 3:** Average number of alleles and observed and expected heterozygosities (H_o_ and H_e_, respectively) in 6 microsatellite loci for each group (sorted by parents or by populations) used in the mating experiments of *Planococcus* *citri* .

	**Sample size**	**Average number of alleles**	**Average H_o_**	**Average H_e_**
**Parent**				
Female	43	**2.3**	0.242	0.284
First male	43	**3.2**	0.380	0.560
Second male	43	**2.5**	0.244	0.245
**Population**				
Agualva, Portugal	27	**2.5**	0.352	0.338
Camarate, Portugal	5	**1.5**	0.445	0.277
Mafra, Portugal	31	**1.8**	0.177	0.138
Silves, Portugal	31	**2.3**	0.268	0.253
Tavira, Portugal	2	**1.3**	0.167	0.167
Xirumi-Serravalle, Italy	33	**2.3**	0.391	0.423

In three crosses we could exclude the second males as a parent since they had unique allele(s) that were not detected in the progeny. In two of these the excluded male was heterozygote in the locus that gave such indication and so the possibility of false exclusion due to null alleles is discarded.

## Discussion

Microsatellite analysis revealed that both males of *P* *. citri* transmitted genes to the progeny of doubly mated females in at least 30% of controlled crosses. This result constitutes the first genetic evidence of polyandry in any scale insect, supporting the behavioral evidence recently published ( [11,12,13]; Silva et al., unpublished data). However, the levels of polyandry in laboratory conditions may not reflect what happens in natural populations [1,35,36]. Further studies are needed to assess the existence of polyandry in field populations of mealybugs. Although the approach described here may allow us to find evidence of multiple paternity in the progeny of pregnant females collected in the field, it would be advisable to use more variable markers since the relatively low level of polymorphism in the used microsatellite loci may not allow efficient paternity assignment in cases where the potential male parents are not known.

The occurrence of mutations, genotyping errors and null alleles has to be taken into account in every study of paternity assignment or exclusion [37,38]. The fact that in our study every allele of each female was detected in the progeny supports the assumption that PCR amplification is not under-amplifying some alleles, although such under-amplification may occur. Also, no different alleles from the ones detected in the parents appeared in the progeny, which indicates that the error rate is low. Genotyping error rates reported in other studies ranged from 0.2 to 15% per locus [39]. In this particular case, in which we tested the occurrence of multiple paternity in very controlled settings, with known genotypes of the mother and of the two potential fathers involved, the chances for misassignment are expected to be very low. For the conclusion of the occurrence of polyandry to be undermined in this study, we would need to assume that a genotyping error occurred in over 30% of the crosses.

Our data also provide genetic evidence for the chromosomal inheritance of *P* *. citri* which was previously based on cytogenetic studies [17]. The fact that some mealybug males were heterozygotes in some loci, i.e., diploid, but only transmitted one of the two alleles, is a clear indication that they are functionally haploid, supporting PGE.

In three of the crosses, only the first male fertilized the eggs, although the two males mated with the female in every cross, which may indicate the occurrence of sperm competition or unviable sperm supply [2,4]. In several other crosses, it was not possible to determine if the second male fertilized the eggs because those males were on average less polymorphic and less heterozygotic than first males and so they had fewer distinct alleles making it impossible to attribute paternity. This alerts us to the bias in the detection of paternity if one male has a genotype different from the female, but the other has not. This type of bias has been described for the detection of extra-pair paternity, which is favoured when the extra-pair males have different genotypes from the females, leading to the erroneous conclusion that extra-pair males are less related to the females [40].

In cases where polyandrous fertilizations were detected using the pool of eggs, we do not know the proportion of descendants fathered by each male. Analysing single-individual genotypes will be essential in following work to address questions about sperm competition, e.g does the order of mating affect the success of fertilization by the males?; what proportion of fertilizations by each male are successful?

Increased fertility and fecundity are among the reported benefits of polyandry (reviewed by [1,3]). However, with regard to mealybugs, Waterworth and colleagues [11] found no relationship between female fecundity and the number of copulations in the three tested mealybug species. We obtained similar results for *P* *. calceolariae* (Silva et al. in preparation). In this context, Waterworth and colleagues [11] suggested that for mealybug males copulation with mated females is expected to be a wasted reproductive effort, unless mechanisms such as sperm precedence are involved. Further studies are needed to clarify this issue. Alternatively, the benefit of multiple mating for mealybug females may be associated with the increment of genetic variability among offspring, which might be especially important in insects whose males are functionally haploid and only transmit half of the genome, i.e., that inherited from the mother. Furthermore, it has been suggested that polyandry might be a way for females to avoid the negative effects of genetic incompatibility, i.e. inbreeding [41]. Due to their spatial aggregation pattern [42] and functional haplodiploidy system [19], mealybugs are expected to present a high risk of inbreeding. Finally, considering that multiple mating might have evolved to avoid fertilization with sperm containing selfish genetic elements [6,7], the presence of “selfish genetic elements”, such as supernumerary B chromosomes and endosymbionts, which might be involved in different genetic conflicts [21], may also favor polyandry in mealybugs.

Chemical control is still the most common control tactic used against pest mealybugs. However, insecticides are often ineffective due to the peculiar biological characteristics of mealybugs, e.g. cryptic behavior, wax body cover, and overlapping generations [10,43]. Pheromone-based management tactics, such as mass trapping, mating disruption, and lure and kill, have been considered sustainable and environmentally friendly alternatives [10,44–46]. Nevertheless, the success of pheromone-based control methods is dependent on the understanding of the mating system of the target insect pest [14,47,48] ⁠. The possible occurrence of polyandry in mealybugs has practical implications for the effectiveness of these control methods. For example, mating disruption is expected to have more impact on mealybug populations with a polyandrous mating system in comparison to monandry. Under effective mating disruption, males have a very low chance of locating a female and mate. Therefore, the rate of mating is limited and that of multiple mating almost nil. In such a situation, in absence of multiple mating, none of the benefits associated with polyandry is expected to occur in those mealybug populations.

The cost-effective approach described here for detecting polyandry, i.e. genotyping pools of eggs instead of single individuals, can be applied in other situations where the number of descendants is very high and would be very expensive and laborious to genotype every single individual. This type of approach has been previously successfully applied to the analysis of the sperm contained within the spermatheca of female insects, trying to detect microsatellite alleles of different males to have an estimate of the number of inseminations but not of the successful fertilizations [35,49].
